# Utilization of the ART approach in a group of public oral health operators in South Africa: a 5-year longitudinal study

**DOI:** 10.1186/1472-6831-9-10

**Published:** 2009-04-21

**Authors:** Steffen Mickenautsch, Jo E Frencken

**Affiliations:** 1 Division of Public Oral Health, University of the Witwatersrand, Johannesburg, South Africa; 2 Nijmegen International Oral Health Unit Nijmegen, Radboud University Medical Centre, Nijmegen, the Netherlands

## Abstract

**Background:**

A significant increase in the proportion of restorations to the number of tooth extractions was reported after the introduction of ART in an academic mobile dental service in South Africa. The changes were ascribed to its less threatening procedure. Based on these findings, ART was subsequently introduced into the public oral health service of Ekurhuleni district in the South African province of Gauteng. This article reports on the 5-year restorative treatment pattern of operators in the Ekurhuleni district, who adopted the ART approach into their daily dental practice.

**Methods:**

Of the 21 trained operators, 11 had placed more than 10% of restorations using ART at year 1 and were evaluated after 5 years. Data, including number of restored and extracted teeth and type of restoration, were drawn from clinical records 4 months before, and up to 5 years after training. The restoration/extraction ratio (REX score) and the proportion of ART restorations to the total number of restorations were calculated. The paired sample t-test and linear regression analysis were applied.

**Results:**

The mean percentage of ART restorations after 1 year was 24.0% (SE 7.2) and significantly increased annually to 42.7% (SE 9.2) after 5 years in permanent dentitions. In primary dentitions the mean percentage of ART restorations after 1 year was 80.6% (SE 4.9) and 72.6% (SE 8.8) after 5 years. The mean REX score before ART training was 0.08 (SE 0.03) and 0.07 (SE 0.04) for permanent and primary teeth, respectively and 0.11 (SE 0.03) and 0.17 (SE 0.05) after 5 years.

**Conclusion:**

Five years after training, ART had been used consistently in this selected group of operators as the predominant restorative treatment used for primary teeth and showed a significant annual increase in permanent teeth. However, this change had not resulted in an increase in the REX score in both dentitions.

## Background

The South African Department of Health conducted a national oral health survey in 1988/9. It covered only urban areas in the country's nine provinces. The authors observed, among others, the need for restorative treatment in the ratio of 2 restorations to 1 extraction [1]. Ten years later a report showed that restorative care in the public oral health services was provided in a ratio of only 1 restoration to 9 extractions [2].

Using the 2001-census data, the dental operator to person ratio in South Africa was found to have been in the order of 1 to 95 727 [3]. Each dental operator in the public oral health services rendered, on average, 4 400 oral treatment procedures per year [2]. These services were provided in 490 full-time and 322 part-time operating dental surgeries [2] and have been described as palliative, demand-driven, and lacking a structured budget and functional concepts [2]. It seemed, therefore, very unlikely that the continuing use of the current traditional rotary-driven restorative treatment regime would lead to attainment of the Department's goal of reducing premature tooth loss within the population, in the foreseeable future [1].

An appropriate alternative to the traditional restorative treatment approach is Atraumatic Restorative Treatment (ART). ART has been developed for managing dental caries and relies on the use of hand instruments for removal of carious tissues and filling of the cleaned cavity and adjacent fissures with a high-viscosity glass ionomer cement [4]. Research has shown high mean survival rates for single-surface ART restorations using high-viscosity glass ionomer cement in both primary and permanent teeth [5]. Single-surface ART restorations in permanent teeth have also been reported to survive longer than comparable restorations produced through the traditional approach using amalgam, after 6.3 years [6].

Because of its independence from electricity and expensive dental equipment, the World Health Organisation (WHO) endorsed the ART approach as appropriate for public oral healthcare services in developing countries such as South Africa [7]. In 1996, ART was introduced into an academic mobile dental service (MDS) in South Africa and reported a significant increase in the proportion of restorations to the number of tooth extractions (REX) score after one year [8]. The changes in the REX score were ascribed to the less threatening procedures of ART. Since then, studies have reported that ART causes less pain [9,10] than traditional procedures do and has been associated with less dental anxiety amongst patients, because it does not involve drilling and injections [11,12]. As an association between reduced patient dental anxiety and reduced operator stress exists [13-16], health authorities of Gauteng province in South Africa assumed that dental operators would choose ART instead of the traditional restorative treatment if they had received training in the use of ART. Therefore, in 2001 all 21 public health dental operators of Ekurhuleni, one of the five districts in Gauteng province, attended a training course in ART. Dental operators in other districts constituted the control group. Although tooth extraction remained the main type of treatment provided in both groups one year after training, ART had been used in 67% of the restorations placed in the primary, and 11% of the restorations in the permanent dentition of outpatients in the study group. However, unlike in the MDS programme [8], the REX score did not significantly increase in either dentition in the study group [17]. Furthermore, only 13 of the 21 trained operators were found to have applied ART frequently thus having integrated the approach into their daily dental treatment routine [17]. As no information is available on the long-term effect of the use of ART in public health services by operators who applied ART after completing the training course, a follow up investigation was carried out. This study aims to report on the 5-year restorative treatment pattern of the 13 operators in a South African public oral health service, who adopted the ART approach into their daily dental practice.

## Methods

### Intervention

Permission to carry out the present study was obtained from the Ethics Committee for Research on Human Subjects (Medical) of the University of the Witwatersrand, Johannesburg, South Africa, under protocol number M00/07/13. Operators were trained in ART according to recommended course standards [18] by a staff member (SM) of the Division of Public Oral Health, University of the Witwatersrand, Johannesburg, in August 2001. The training was conducted during a 3-day workshop. Lectures at the Dental School on the first day were followed, on days 2 and 3, by clinical training on selected patients at a primary healthcare clinic in an informal settlement south of Johannesburg. Lectures contained information on (dis)-advantages of ART, its clinical indication, successes and failures of ART restorations and sealants, selection of materials and instruments, hand-mixing of glass ionomer, clinical procedures and management of failed restorations. Operators received copies of the lectures and the ART manual [19]. Contrary to recommendation, no pre-clinical training in the use of ART was given on extracted teeth. Clinical training consisted of demonstration of the use of ART by the trainer, followed by supervised ART treatment of carious lesions by operators. A workshop was attended by groups of 4–6 participants operating in pairs: one carried out the treatment while the other provided chair-side assistance. The functions were alternated for the treatment of successive patients. Each operator restored between 3 and 10 cavities in the 6 – 15-year-old children selected.

The operators received no coaching or support from the health and university authorities after the training.

### Evaluation

Information concerning the number of restored and extracted teeth and type of restoration per dentition was collected in 2002 from dental clinic records covering the 4 months preceding the ART training (April to July 2001) and 12 months after training (August 2001 – July 2002) and, in 2006, for the period from August 2002 to July 2006. The dental operators did the recording. The number of ART and conventional restorations and tooth extractions for both primary and permanent teeth per operator were calculated, by hand from the clinic record books, by the principal investigator and a fieldworker. The dental records formed the basis for calculating the ratio of number of restorations to number of extractions (REX score) and the proportion of ART restorations to the total number of restorations (%ART).

### Statistical analysis

All data were entered into the computer and checked for accuracy before the oral statistician of the College of the Dental Sciences of the Radboud University Nijmegen, the Netherlands, conducted the statistical analysis, using SPSS statistical software. This study follows a retrospective longitudinal design, with the operator as the unit of investigation. A linear regression model was used to estimate the time effect for each dentist for both the proportion of ART restorations and that for the REX score as dependent variables and year as independent variable. From the time effect thus found per dentist, the mean and standard error (SE) of the overall time effect (both for proportion ART restorations and REX score) was calculated. Statistical significance was set at α = 0.05.

## Results

Of the initial 21 dental operators, 8 operators did not use ART after training [17]. Two further operators had left the services during the period from 2002 to 2006. The remaining 11 operators adopted ART into their daily dental practice and were followed longitudinally. These consisted of 7 females and 4 males, 8 of whom were dentists and 3, dental therapists. The mean age was 41 years (SD = 9.5). In 2006, operators had graduated on average 18 (SD = 7.4) years previously and worked in their current posts on average for 14 (SD = 3.6) years.

**Figure 1:**
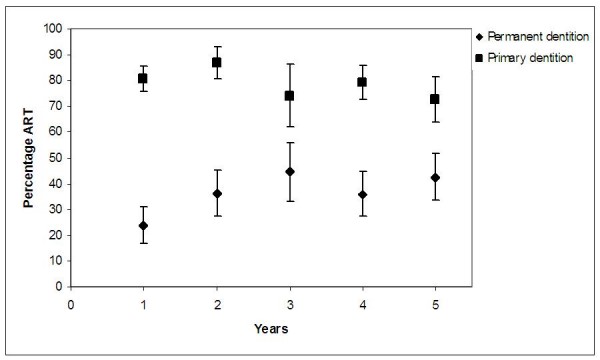
**Percentages of ART restoration (%ART) and Standard Error (SE) for the primary and permanent dentition by year of investigation**.

The percentage of ART restorations and standard error of the total number of permanent and primary restorations placed over the 5-year period are shown in Figure 1. The mean percentage of ART restorations in permanent dentition after 1 year was 24.0% (SE 7.2) and increased to 42.7% (SE 9.2) after 5 years. This increase was statistically significant (p = 0.02). The percentage of ART restorations in primary dentition after 1 year was 80.6% (SE 4.9) and 72.6% (SE 8.8) after 5 years.

**Figure 2:**
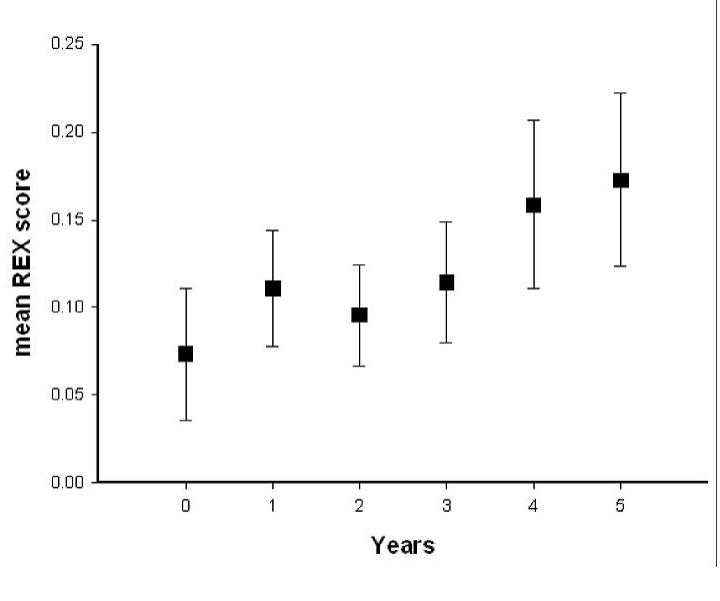
**Mean REX scores and Standard Error (SE) for primary dentitions before ART training (Year = 0) and 5 years after ART training**.

**Figure 3:**
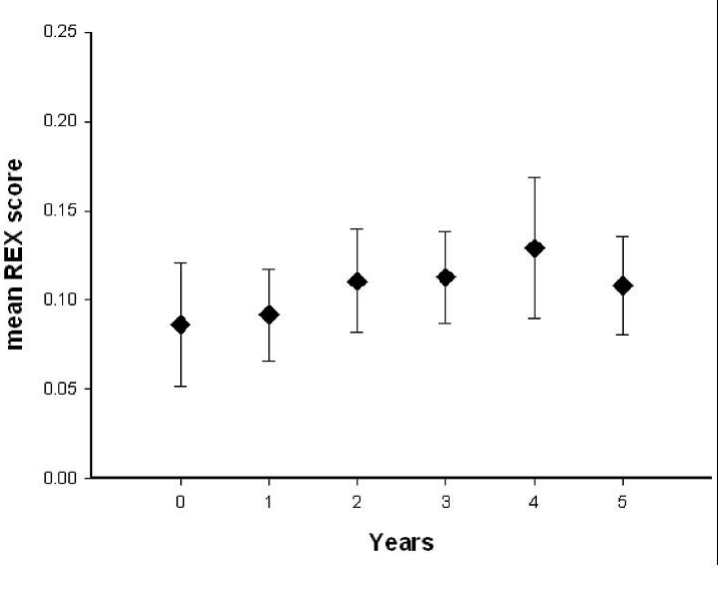
**Mean REX scores and Standard Error (SE) for permanent dentitions before ART training (Year = 0) and 5 years after ART training**.

The mean REX scores and standard error from before the introduction of ART training to 5 years after training in primary and permanent dentition are shown in Figures 2 and 3, respectively. The mean REX score before ART was introduced was 0.08 (SE 0.03) and 0.07 (SE 0.04) for permanent and primary teeth, respectively. Five years after ART training, the mean REX score was 0.11 (SE 0.03) for permanent and 0.17 (SE 0.05) for primary dentitions. No time effect was observed for the mean REX scores in permanent (p = 0.59) and primary (p = 0.24) dentition from before, to 5 years after, the ART training.

## Discussion

This investigation reports on the use of ART by a selected number of dental operators in a provincial public oral health service, 5 years after they were trained. The preceding study, carried out one year after the training, was aimed at assessing the effect of the ART training and had, therefore, included a control group [17].

As the investigation is a selective follow-up to an earlier published study [17], it has inherited some study design shortcomings. These include a potential recall bias related to the fact that treatment data were sometimes recorded by staff at the end of the day and not immediately after completion of the treatment. Recording in this way is, however, common practice in South Africa. A further shortcoming is that evaluator blinding was impossible. It would have required the employment of an outside evaluator totally ignorant about the ART training, for a period of 5 years. Such a requirement is very difficult to meet, considering the publications on ART, and an evaluator was, therefore, not available.

Only 11 from the originally 21 operators adopted ART into their daily dental practice. The reasons may be related to barriers of ART adoption, which were investigated and reported elsewhere: lack of a sustained supply of materials for placing ART restorations; lack of adequate operator time, due to high patient load/workload, lack of patient cooperation due to dental anxiety; lack of leadership and guidance by healthcare management, negative attitudes of patients towards receiving restorative care; insufficient chair-side assistance and negative operator attitude towards using ART [20].

The results of this study showed no statistically significant difference in the proportion of ART restorations in relation to the total number of restorations placed in primary dentitions over the five-year study period, but it did in permanent dentitions. Dental operators had maintained their relatively high level of ART utilisation in primary teeth from year 1 to year 5 and increased it in permanent teeth. It appears, therefore, that in this selected group a single ART training course had resulted in a sustained shift in the restoration pattern, particularly in primary teeth; from predominantly conventional restorative treatment, using rotary instruments, to ART. This indicates that these operators preferred ART as the mode for treating children.

Despite the increase in ART restorations in both dentitions, no statistically significant changes in the mean REX score over the period preceding ART training to 5 years following it could be observed. This may indicate that, although dental operators may prefer to use ART in many cases, this preference was not strong enough to motivate them to use ART at a higher frequency than conventional restorative treatment. It may also indicate an increase in tooth extractions.

As epidemiological data in South Africa have shown a need for twice as many tooth restorations as extractions [1], expressed as mean REX score of 2.0, it is obvious that the introduction of ART has not resulted in achieving this national goal. The REX scores of 0.11 and 0.17 for permanent and primary teeth respectively in the present study indicate that many cavities were not restored.

Reasons for this situation may be related to the barriers of ART adoption in South African public oral health services [20]. A critical shortage of dental operator posts in the South African public oral health service has also been reported [2,21]. This, combined with an increasing number of patients seeking care at public dental clinics creates a constant high patient load/workload [2,21] and thus limits time needed for the operator to address patients' restorative needs.

As in many African countries, demands by patients for tooth extractions in South Africa's public oral health clinics are higher than for tooth restorations [22]. Patients, particularly from a low socio-economic background, seek care only when they have severe toothache, which has to be treated by extraction of the decayed tooth. Even if a cavitated lesion can be treated with a restoration, few patients honour the appointment made. The situation was studied in Tanzania. Poor communication between the dental practitioner and dental outpatients was the major barrier why people did not receive restorative care. The outpatients did not know that a tooth could be restored [23] and the dental practitioner did not inform them that such a treatment was possible [24]. Even if good communication lines between the patients and dental practitioners are established, it does not imply that the percentage of teeth restored will increase. In South Africa as in other African countries, patients often have to travel long distances to a dental clinic and when arrived, have to queue for long hours until they are attended to. A recall visit is then no option and a painful tooth that can be saved through a restoration is extracted. This happens even though free oral health services are offered in public oral health clinics. Patients have to pay for transport to the clinics and that further results in very low recall compliance amongst patients in public oral health services.

## Conclusion

Five years after training, ART had been used consistently by the investigated selected group of dental operators: it was not considered a novelty to be used for only a short time. The ART approach was the predominant restorative treatment used in primary teeth and showed a significant annual increase in permanent teeth. However, this sustained use had not resulted in a statistically significant increase in the REX score in primary and permanent dentitions.

## Competing interests

The authors declare that they have no competing interests.

## Authors' contributions

SM conducted the data collection of this study and contributed to the writing of the manuscript. JF initiated the study and contributed to the writing of the manuscript.

## Pre-publication history

The pre-publication history for this paper can be accessed here:



## References

[B1] du Plessis JB, Carstens IL, Rossouw LM, Olivier I, van Wyk PJ (1994). The dental caries status of the urban population in the major metropolitan areas of the Republic of South Africa. National Oral Health Survey: South Africa 1988/89.

[B2] Gugushe T (1998). Compulsory Community Service Audit For Dentists In South Africa.

[B3] Census 2001 (2003). Census In Brief: Report No 03-02-03 (2001).

[B4] Frencken JE, Pilot T, Songpaisan Y, Phantumvanit P (1996). Atraumatic restorative Treatment (ART): Rationale, Technique and development. J Public Health Dent.

[B5] van't Hof MA, Frencken JE, van Palenstein Helderman WH, Holmgren CJ (2006). The atraumatic restorative treatment (ART) approach for managing dental caries: a meta-analysis. Int Dent J.

[B6] Frencken JE, Taifour D, van't Hof MA (2006). Survival of ART and amalgam restorations after 6.3 years. J Dent Res.

[B7] World Health Organisation (1998). Atraumatic Restorative Treatment (ART) For Tooth Decay Geneva.

[B8] Mickenautsch S, Rudolph MJ, Ogunbodede EO, Frencken JE (1999). The impact of the ART approach on the treatment profile in a mobile dental system (MDS) in South Africa. Int Dent J.

[B9] Rahimtoola S, van Amerongen E, Maher R, Groen H (2000). Pain related to different ways of minimal intervention in the treatment of small caries lesions. ASDC J Dent Child.

[B10] Louw AJ, Sarvan I, Chikte UME, Honkala E (2002). One-year evaluation of atraumatic restorative treatment and minimum intervention techniques on primary teeth. SADJ.

[B11] Schriks MC, van Amerongen WE (2003). Atraumatic perspectives of ART: psychological and physiological aspects of treatment with and without rotary instruments. Community Dent Oral Epidemiol.

[B12] Mickenautsch S, Frencken JE, van't Hof MA (2007). Atraumatic restorative treatment and dental anxiety in outpatients attending public oral health clinics in South Africa. J Public Health Dent.

[B13] Humphris GM, Peacock L (1993). Occupational stress and job satisfaction in the community dental service of north Wales: a pilot study. Community Dent Health.

[B14] Möller AT, Spangenberg JJ (1996). Stress and coping amongst South African dentists in private practice. J Dent Assoc S Afr.

[B15] Newton JT, Gibbons DE (1996). Stress in dental practice: a qualitative comparison of dentists working within the NHS and those working within an independent capitation scheme. Br Dent J.

[B16] Moore R, Brødsgaard I (2001). Dentists' perceived stress and its relation to perceptions about anxious patients. Community Dent Oral Epidemiol.

[B17] Mickenautsch S (2007). The Impact Of The ART Approach On The Treatment Pattern In A Public Oral Health Service In South Africa.

[B18] Frencken JE, Holmgren C (2000). Atraumatic Restorative Treatment (ART) For Tooth Decay A Global Initiative 1998 – 2000: How To Organize And Run An ART Training Course.

[B19] Frencken J, Phantumvanit P, Songpaisan Y, Pilot T, van Amerongen E (1997). Manual For The Atraumatic Restorative Treatment Approach To Control Dental Caries.

[B20] Mickenautsch S, Frencken JE, van't Hof MA (2007). Barriers for the implementation of the atraumatic restorative treatment approach in public oral health service. J Appl Oral Sci.

[B21] Bhayat A, Cleaton-Jones P (2003). Dental clinic attendance in Soweto, South Africa, before and after the introduction of free primary dental health services. Community Dent Oral Epidemiol.

[B22] Gilbert L, van Rooy HK, Snyman WD, Olivier I, van Wyk PJ (1994). Perceived need in relation to epidemiological determined need for dental treatment in South Africa. National Oral Health Survey: South Africa 1988/89.

[B23] Kikwilu EN, Frencken JE, Mulder J, Masalu JR (2009). Barriers to restorative care as perceived by dental patients attending government hospitals in Tanzania. Community Dentistry Oral Epidemiology.

[B24] Kikwilu EN, Frencken JE, Masalu JR, Mulder J Barriers to restorative care as perceived by dental practitioners in Tanzania. Community Dent Health.

